# Myasthenia and bipolar disorder: a case report and review of literature

**DOI:** 10.1192/j.eurpsy.2022.1155

**Published:** 2022-09-01

**Authors:** B. Emna, R. Kammoun, M. Kroui, F. Ellouz

**Affiliations:** 1 Razi hospital, Psychiatry G, manouba, Tunisia; 2 Razi hospital, Psychiatry G, denden, Tunisia

**Keywords:** bipolar disorder, comorbidity in bipolar disorder, myasthenia, auto-immune disorder

## Abstract

**Introduction:**

The association between somatic diseases and bipolar disorder (BD) have been described especially for auto-immune diseases.

**Objectives:**

Through a case study and a review of literature we are going to describe a comorbidity of BD and myasthenia.

**Methods:**

Starting from a case report, we conducted a literature review on “PubMed”, using a key word “myasthenia and bipolar disorder”

**Results:**

The patient AJ, 57 years old, married, mother of 5 children; 4 sons and 1 daughter who also has BD. She is illiterate and a full-time mother. she has high blood pressure, a congestive gastropathy and hemorrhoids. She has been diagnosed with BD in 1987 (at 21 years old) and mainly had depressive episodes. She was put on Amitriptyline, carbamazepine, long-acting neuroleptics and benzodiazepines. Since 2006 the patient has been reporting persisting myasthenia even when she was euthymic. In 2009, she was hospitalized for persistent headaches, pain and a decrease in visual acuity in the right eye. An ophthalmoscopy, a cranial CT-scan and an MRI were performed with no anomalies. Then a fluctuant ptosis and an intense fatigability appeared. She then was hospitalized in a neurology ward where she was diagnosed with myasthenia. Changes in her treatment had to be made. Carbamazepine was switched to valproic acid, amitriptyline was switched to fluoxetine. And benzodiazepines were stopped.

**Conclusions:**

It’s important to pay close attention to somatic diseases in our patients in order to insure appropriate medical care. Also, the association between somatic disorders and BD can be an interesting lead in elucidating the etiopathogeneses of BD.

**Disclosure:**

No significant relationships.

